# A Preliminary Report on Disordered Speech with Deep Brain Stimulation in Individuals with Parkinson's Disease

**DOI:** 10.4061/2011/796205

**Published:** 2011-10-16

**Authors:** Christopher Dromey, Suzy Bjarnason

**Affiliations:** Department of Communication Disorders, Brigham Young University, Provo, UT 84602, USA

## Abstract

Deep brain stimulation (DBS) of the subthalamic nucleus (STN) has proven effective in treating the major motor symptoms of advanced Parkinson's disease (PD). The aim of this study was to learn which laryngeal and articulatory acoustic features changed in patients who were reported to have worse speech with stimulation. Six volunteers with PD who had bilateral STN electrodes were recorded with DBS turned on or off. Perceptual ratings reflected poorer speech performance with DBS on. Acoustic measures of articulation (corner vowel formants, diphthong slopes, and a spirantization index) and phonation (perturbation, long-term average spectrum) as well as verbal fluency scores showed mixed results with DBS. Some speakers improved while others became worse on individual measures. The magnitude of DBS effects was not predictable based on the patients' demographic characteristics. Future research involving adjustments to stimulator settings or electrode placement may be beneficial in limiting the negative effects of DBS on speech.

## 1. Introduction

A common sign of Parkinson's disease (PD) is hypokinetic dysarthria [1]. Typical speech characteristics include a weak, breathy voice, abnormal prosody, variability in rate, and imprecise movements of the articulators [2]. In addition, individuals with PD frequently have reduced facial animation and limited mobility of their oral musculature [3]. 

Prior to the 1960s, thalamotomy and pallidotomy surgeries were performed to treat symptoms of advanced PD, but reliance on these operations decreased dramatically when levodopa became widely available [4]. However, it subsequently became clear that levodopa, when used for an extended period of time, can cause problems of its own, such as drug-induced dystonia and dyskinesia [5]. Many patients also experience *on-off effects*, or periods when the motor benefits of levodopa are stable and then suddenly deteriorate [6].

A 1987 publication by a team in Grenoble sparked a renewed interest in surgical approaches to treating movement disorders [7], specifically through deep brain stimulation (DBS). In this procedure, which has evolved significantly since the early 1990s, electrodes are permanently implanted into the thalamus, globus pallidus, or subthalamic nucleus (STN) and the signals from an implanted pulse generator are used to influence neural activity in the basal ganglia circuitry and its output via the thalamus to the motor cortex. DBS is often preferred over tissue ablation procedures because negative side effects of DBS can be mitigated by adjusting the parameters of the stimulator or by removing the hardware [8], whereas problems that may result from thalamotomy and pallidotomy lesions are permanent.

Despite the potential for occasional surgical complications, DBS of the STN has become the preferred treatment method for many patients with advanced PD because it improves the major symptoms of the disease more effectively than when the globus pallidus is stimulated [9]. Patients who receive STN-DBS can significantly decrease the levodopa dosage they need to control their symptoms [8] and thus lessen the severity of the drug side effects.

Research into the effects of DBS on speech has yielded mixed results. Some studies have shown that DBS in PD leads to improvements in general motor function that are far more substantial than those in speech [10, 11]. Farrell et al. [3] found that individuals with PD who had surgery (thalamotomy, pallidotomy, or DBS) displayed a marked reduction in Hoehn and Yahr staging of PD scores when compared with a nonsurgery PD group, but they found that there were no significant changes in their speech. One long-term international study of 69 patients receiving bilateral STN or pallidal stimulation reported that speech disturbances were relatively frequent, with severe impairment in five of the patients [12]. Other reports have also revealed that speech can be negatively affected with surgical intervention [13]. Gentil et al. suggested that “speech may be worsened with STN stimulation when using excessively high or too low stimulation parameters and in case of incorrect location of deep brain electrodes in the STN” [14, page 194]. Others have concluded that higher amplitude settings can result in a decrease in speech intelligibility [15]. 

Gentil et al. [14] reported that all participants in their study who received stimulation of the STN demonstrated improvements in speech, whereas the speakers who had mild or moderate dysarthria without stimulation were negatively affected by stimulation of the ventral intermediate nucleus of the thalamus. Wang et al. [9] showed that bilateral stimulation in the subthalamic nucleus had some positive effects on speech. However, they found no changes in speech with unilateral stimulation. Gentil et al. [16] found that bilateral stimulation of the subthalamic nucleus improved the strength and precision of articulator movements for nonspeech behaviors in individuals with PD. However, the movements of these same structures in speech are far more subtle and complex, and Montgomery [17] suggested that attempts to explain speech motor control on the basis of our understanding of limb movement regulation may be unsuccessful. He suggested a potential resonance mechanism whereby DBS with higher frequency pulses could lead to improvements in the relatively simple movements involved in limb function, while negatively impacting the bulbar circuitry involved in speech production.

In summary, research suggests that DBS of the subthalamic nucleus can improve motor functioning for many patients, but further research needs to be conducted to determine the specific effects of this treatment on speech. Because both positive and negative speech effects have been reported to follow DBS, the present investigation focused on a group of patients who were reported by medical personnel to have worse speech with STN stimulation. The surgical team had expressed interest in learning more specifically which aspects of speech became worse with DBS so that future patients might potentially benefit from refinements in the intervention. The study involved the analysis of several speech acoustic measures in both on- and off-stimulation conditions in order to evaluate phonatory and articulatory performance, as well as an index of verbal fluency. The goal was to learn whether the impact of DBS would be different across the subsystems of speech or whether a more consistent, negative effect would be found in this group of speakers.

## 2. Method

### 2.1. Participants

The participants were six patients aged 48–79 years, who had mild to moderate idiopathic PD (see Table 1 for demographic details). All participants had been implanted with bilateral electrodes in the STN at the University of Utah Medical Center. All participants volunteered to be in the present study and signed an IRB-approved informed consent document. They were referred for participation in the study because the neurology staff who performed the DBS programming had observed worse speech with stimulation than without. It was reasoned that a deterioration in speech performance that was apparent to individuals outside the field of communication disorders would be significant enough to warrant further investigation by way of the acoustic and perceptual measures applied in the current study. Thus, a formal diagnosis of dysarthria by a speech-language pathologist was not an inclusion criterion. The patients had not received any speech/language therapy prior to their referral. Information regarding the acoustic or perceptual speech status of the patients prior to electrode implantation was not available because the patients were referred to us only after their DBS had been in place for at least six months. However, the primary aim of the study was to quantify several acoustic aspects of speech deterioration rather than to track pre- to postsurgical change.

### 2.2. Speaking Tasks and Speech Sample

All participants were optimally medicated at the time of the study, which took place at least six months after the surgery. A minimum recovery period of six months was used because the stimulation parameters of the pulse generator had been clinically programmed by that time, and thus speech quality and limb function would be generally stable. After recording participants in the stimulation-on condition, the stimulator was turned off; subsequent recordings in the off condition took place one hour later to ensure that the effects of stimulation would have diminished.

Participants read the sentence “The boot on top is packed to keep” to elicit productions of the corner vowels /i/, /*ɑ*/, /u/, and /æ/ in a consonant-vowel-consonant context. This sentence was selected because it had a stress pattern typical of natural English speech, and each word containing a corner vowel received stress. The sentence “The boy gave a shout at the sight of the cake” was also read to elicit the diphthongs /*ɔɪ*/, /a*ʊ*/, /a*ɪ*/, and /e*ɪ*/. Each participant repeated both sentences five times. These tasks were selected in order to allow the computation of formant measures reflecting articulatory activity. The speakers also read the first six sentences of the Rainbow Passage [18] to allow a perceptual assessment of speech quality as well as the computation of a long-term average spectrum. Alternating motion rate (AMR) syllables (/p/, /t/, and /k/) were recorded in order to allow the calculation of an index of spirantization, given the occurrence of this articulatory deficit in some speakers with hypokinetic dysarthria [19]. This was followed by a one-minute verbal fluency task in which participants were asked to list all of the words they could think of that started with the letter *r*, *w*, or *p*. These letters have been used in a previous investigation of DBS effects on word retrieval [20]. The initial letter of the word was selected at random, and the letter selected for each participant was different in the on and off conditions. This task was completed in order to learn whether DBS influenced a simple word-finding task. Finally, participants were instructed to take a deep breath and then sustain /*ɑ*/ for as long as possible. This task allowed the computation of vocal perturbation measures.

### 2.3. Instrumentation

During each of these tasks, the acoustic signal was recorded into a Dell laptop computer via a headset microphone (AKG C-420) with a mouth-to-microphone distance of approximately 5 cm. A Tascam US-122 USB interface was used to digitize the acoustic signal from the microphone.

### 2.4. Data Analysis

To obtain measures of verbal fluency, a count was taken of the number of words each participant was able to produce in a 30-second period in each condition. Any nonwords that were produced were not included in the total. Both a strict count (no proper nouns allowed) and a lenient count (proper nouns allowed) were made, because some speakers included proper nouns while others did not. Verbal fluency was measured as a simple index of word-finding abilities.

Measures of jitter, shimmer, and harmonics to noise ratio (HNR) were computed with Praat 4.5.18 software [21] from a 2-second window that began 2 seconds into the sustained vowel recording. A 1-second vowel sample was used for speaker M5, who was not able to sustain phonation long enough to match the minimum 4-second duration that the other speakers produced. Phonatory function has previously been reported for STN-DBS [10] and was included in the present study to examine the impact of DBS on laryngeal activity.

Using TF32 software [22] the long-term average spectrum (LTAS) was calculated for the Rainbow Passage that was read by each participant. This measure was of particular interest because Dromey [23] reported that statistical measures of the LTAS shape, referred to as *spectral moments* of the LTAS appear to be sensitive to changes in voice quality in speakers with hypokinetic dysarthria. The first two spectral moments (mean and standard deviation) of the LTAS were used to indirectly assess the voice quality during connected speech.

Spirantization during the AMR task was assessed by computing a vowel to stop closure intensity ratio based on the root mean square (RMS) trace in TF32. This was done by measuring the amplitude of a 30 ms segment in the middle of the /*ə*/ vowel, as well as a 30 ms segment in the middle of the preceding stop closure for each of 10 syllables with and without stimulation. The 30 ms window was chosen to allow the measurement of energy even in a brief closure or vowel, as can be occasionally observed in speakers with PD during AMR tasks. A lower vowel to stop closure ratio would reflect greater spirantization, because frication noise during the intended closure would elevate the stop gap RMS level. Since spirantization has been associated with hypokinetic dysarthria [19], it was reasoned that this index may reveal changes in articulatory precision in response to DBS.

To determine the effect of DBS on the speed and extent of tongue movement in the productions of diphthongs, the segments /*ɔɪ*/, /*ɑʊ*/, /*ɑɪ*/, and /e*ɪ*/ were extracted from five repetitions of the sentence *The boy gave a shout at the sight of the cake* using Praat. The slopes of the first two formants of the diphthongs were computed in each condition, after which the values across the five repetitions were averaged together to obtain a mean slope for F1 and F2 for each diphthong for each participant.

From the sentence *The boot on top is packed to keep*, the first and second formant frequencies of the corner vowels were measured using Praat for each of the five repetitions, which were then averaged. Vowel space area was calculated using Matlab 7.1 [24]. The F1 and F2 averages were plotted in Matlab to create a vowel quadrilateral. The quadrilateral area (in Hz^2^) was calculated using the Matlab polygon area function to determine total vowel space area under each stimulation condition. Vowel space area has been reported in previous studies of dysarthria [25], and the goal in the present study was to learn whether it would be influenced by DBS.

Perceptual ratings of dysarthria severity were made by three first year graduate students in speech-language pathology who had limited experience with dysarthric speech. The raters listened to the sentences that were used for the formant measures and also to the Rainbow Passage. The two spoken tasks in both on and off conditions for the six speakers resulted in the rating of 24 samples. Six of the samples were randomly repeated to allow an estimation of intrarater reliability. The listeners were blind to the purpose of the study and the speaking condition. All samples were presented in the same randomized order for all listeners. They were asked to slide a computer marker with a mouse along a continuum that was labeled on the left as normal and on the right as severely dysarthric. This visual analog scale yielded a score between 0 and 100, with higher numbers representing greater severity. The judges were asked for a single, global rating of speech severity, rather than an evaluation of the individual aspects of speech such as phonatory quality, prosody, and articulatory accuracy. Thus it was reasoned that less experienced listeners would be suitable for the rating task, given that previous studies have reported that listener training and experience are not consistently associated with greater rater reliability [26, 27].

## 3. Results

The small number of participants in the current study makes it difficult to generalize the findings to a larger population. Because of this, no group inferential statistics were used. Descriptive statistics for individual speakers are presented in the data tables to reflect their performance on the different tasks in the on and off conditions. On the basis of the speakers' referral to the study, it would be anticipated that many of the acoustic indices would reveal poorer performance with stimulation. However, this was not always the case.

### 3.1. Verbal Fluency

The verbal fluency counts are reported in Table 2. The patterns in the data were consistent for both the strict and the lenient criteria, showing that four of the six participants (F1, M4, M5, and M8) were able to produce more words in the off condition than in the on condition. Thus for these speakers, DBS appeared to make word finding more difficult.

### 3.2. Perturbation

Table 3 reports the vocal perturbation data, which showed poorer laryngeal performance for three of the speakers in the on condition and for three in the off condition. Poorer performance was reflected in higher jitter and shimmer percent scores and a lower harmonics-to-noise ratio (HNR). For some of the participants (e.g., M9) the differences were subtle, whereas other speakers (F1, M4) experienced a larger effect from stimulation. For speaker F1, STN stimulation resulted in much higher jitter values and a substantial drop in HNR. These changes suggest that phonation was more irregular and unsteady with DBS on. For speaker M4 shimmer increased markedly with stimulation and HNR decreased. As with speaker F1, these changes for speaker M4 reflect poorer vocal function with stimulation.

### 3.3. Long-Term Average Spectrum

As shown in Table 4, the spectral mean for the LTAS in the reading passage was lower in the on than in the off condition for four of the participants (F1, M8, M9, and M10), indicating weaker energy in the higher frequencies when they were receiving STN stimulation.

### 3.4. Spirantization

Table 5 shows the ratios of vowel intensity to stop closure intensity for the syllable repetition tasks. If the perception of worse speech with stimulation were related to the extent of spirantization, then a low ratio would be expected when the level of noise during the stop closure increases for spirantized productions, where frication replaces the relative silence of the stop. A higher value reflects reduced spirantization because the vowel has a greater relative intensity than the stop closure. Three of the six participants (F1, M4, and M10) demonstrated a higher ratio for all three syllables with stimulation on, and one participant (M5) had a higher ratio for only two of the syllables with stimulation. Two participants performed better with stimulation off, one showing a higher ratio for two syllables (M8) and the other (M9) exhibiting a much higher ratio for all three syllables.

### 3.5. Formant Slopes

**Figure 1:**
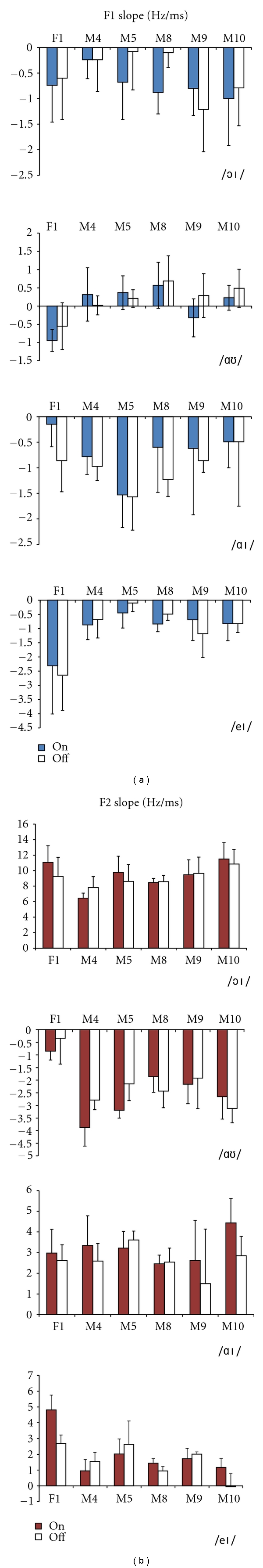
Mean and standard deviation of the first and second formant slopes for all repetitions of the four diphthongs in the on- and off-stimulation conditions for each speaker. In each panel the *x*-axis lists the six speakers and the *y*-axis shows the diphthong slope in Hz/ms.

The slope values (transition extent in Hz divided by transition duration in ms) for F1 and F2 for the diphthongs /ɔɪ/, /ɑʊ/, /ɑɪ/, and /eɪ/ are shown in Table 6; these findings are also graphed in Figure 1. It would be anticipated that the perception of poorer speech with stimulation might be associated with reduced formant slopes, since this measure is reflective of the rate and extent of tongue movement during articulation. When comparing F1 slope across stimulation conditions, one participant (M5) had an increase in slope for three of the four diphthongs with stimulation on, and three participants (F1, M4, and M8) showed a slope increase for two out of four diphthongs with stimulation on. The two remaining participants (M9, M10) appeared to perform more poorly with stimulation, as they only demonstrated greater F1 slopes in the on condition for one diphthong. Thus, with 24 total diphthong productions (four diphthongs × six participants), stimulation resulted in an increase in F1 slope for 11 of the tokens, no change in the slope for three diphthongs, and a decrease in slope for 10 diphthongs.

The results for F2 slope were also quite variable. Stimulation resulted in an increase in F2 slope for all four diphthongs for one participant (F1) and three out of four diphthongs for another (M10). Three participants (M4, M5, and M9) were equally divided across conditions, with an increase in slope for two of the diphthongs with stimulation on and an increase in slope for the other two diphthongs with stimulation off. The remaining participant (M8) only exhibited a greater F2 slope for one diphthong in the stimulation-on condition. Therefore, 14 of the 24 diphthongs produced had a steeper F2 slope with stimulation, while the F2 slope for 10 diphthongs was greater without stimulation.

### 3.6. Vowel Space Area

**Figure 2:**
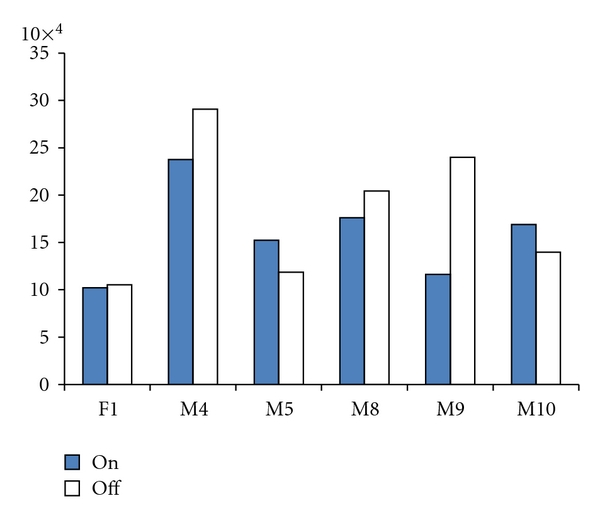
Mean vowel space area (Hz^2^) for all repetitions of the corner vowels in the on and off stimulation conditions for each speaker.

The vowel space areas computed from the average F1 and F2 of the corner vowels /u/, /ɑ/, /æ/, and /i/ for each participant with and without stimulation are presented in Table 7 and Figure 2. A lower number for this measure would be reflective of a smaller acoustic working space for vowels, and thus poorer articulatory performance. The data reveal that four of the six participants (F1, M4, M8, and M9) had a smaller vowel space area in the on condition when compared to the off condition.

### 3.7. Perceptual Ratings

The three listeners who rated dysarthria severity had an average intrajudge reliability of *r* = .93. Interjudge reliability was tested with SPSS 18 and yielded an intraclass correlation coefficient of  .745 for single measures and  .898 for average measures (*F* = 9.78,  *P* < .001). The ratings for the reading passage and the acoustic analysis sentences are presented in Table 8. The general pattern showed an increase in dysarthria severity with stimulation for the reading passage, and, with one exception, the same was true for the acoustic analysis sentences. Speaker M10 was perceived by the listeners to have normal speech for both tasks in each stimulation condition.

## 4. Discussion

The purpose of the study was to investigate the effects of STN-DBS on the speech of individuals with PD who were reported to speak more poorly with stimulation. The objective measures revealed a mix of positive and negative speech changes.

### 4.1. Verbal Fluency

Stimulation of the STN resulted in poorer verbal fluency performance for four out of six participants. The present results are consistent with the findings of others [28, 29], who have reported reduced verbal fluency scores with STN-DBS. Similarly, Saint-Cyr et al. [30] found poorer verbal fluency performance with STN-DBS that remained below presurgical levels a year after implantation. A study by Jahanshahi et al. [31] reported no significant changes in either phonemic or semantic verbal fluency scores in patients with either STN or pallidal stimulation. On the other hand, Wojtecki and collaborators [32] reported that verbal fluency improved with DBS stimulation at a low frequency (10 Hz) and suggested that this rate of stimulation may be beneficial for basal ganglia circuits projecting to frontal cortical regions. 

It is possible that the speakers were able to list words beginning with a particular letter more easily than another. The random letter assignment (p, r, or w) resulted in the letter “p” being used more often than the others in the off condition and “r” in the on condition; a systematic counterbalancing of the letters may have potentially yielded different results.

### 4.2. Perturbation

Many individuals with PD experience disordered laryngeal function [2]. If DBS were to affect the weak, breathy voicing often reported in the literature, it could be anticipated that harmonics-to-noise ratio and traditional perturbation measures might reflect such changes. The equally split results—three improved and three worsened with stimulation—suggest important differences in the way individual speakers respond to DBS. The direction of change in vocal function measures with stimulation did not appear to be linked to higher or lower levels of perturbation in the off condition. In other words, the degree of dysphonia did not predict whether stimulation would make the voice better or worse on these measures. A recent study by Hammer and colleagues [33] suggested that high-frequency stimulation of the STN can lead to respiratory overdrive and excessive vocal fold adduction, which may be reflected in higher perturbation values.

### 4.3. Long-Term Average Spectrum

The reduced spectral mean of the LTAS during stimulation for four of the participants may reflect a weaker upper harmonic structure. Dromey [23] reported a lower spectral mean for speakers with PD compared with controls. Thus, for the four speakers in the present study the stimulation may have increased the severity of their hypophonia, although for three of them the effect was modest. Notably, M9, who had the most subtle changes in perturbation, showed the greatest change in the LTAS measures. It may thus be speculated that these two indices of vocal activity are reflective of different changes in phonatory behavior.

### 4.4. Spirantization

Previous studies have documented the presence of spirantization in the speech of individuals with PD [19]. In the present study, a lower vowel to stop amplitude ratio would reflect more severe spirantization. The stimulation-related changes in the present dataset show that individuals differed markedly in the effect of STN-DBS on this measure of consonant articulation. This index may be potentially valuable in quantifying articulatory change in a computationally straightforward way in this population. What is harder to infer from the present data is the physiologic mechanism underlying the findings. Because this index relies on a measure of vowel amplitude as well as air leakage during stop closure, it can be influenced both by vowel weakness and spirantization at the place of articulation, rather than being a measure of consonant precision alone.

### 4.5. Formant Slopes

Forrest et al. [34] found that the formant transitions of speakers with PD were smaller than those of healthy geriatrics. Perceived worsening of speech with stimulation might be expected to result in smaller formant transitions but the present data reflect patterns of both increases and decreases. Poluha et al. [35] hypothesized that a reduction in rigidity and bradykinesia from PD patients' use of levodopa would permit faster articulatory changes and thus result in a greater F2 slope. The mixed findings in the present study suggest that DBS may have had this effect on a subset of the speakers, but that others did not benefit in the same way.

It should be noted that these formant slope findings do not align in a straightforward way with the results of vowel space area analysis. Some speakers showed an increase on one measure but a decrease on the other when the STN was stimulated.  Although both measures indirectly reflect the extent and/or rate of tongue movement during speech, vowel space area is a measure of an individual's acoustic working space while formant slopes are an indication of transitions from the onset to the offset in a diphthong. It would be valuable in future research to learn whether in a large sample of healthy or dysarthric speakers there is a robust correlation between greater vowel space area and steeper diphthong slopes.

### 4.6. Vowel Space Area

Tjaden and Wilding [25] reported that reduced vowel space area is characteristic of individuals with PD as a result of smaller displacements of the articulators during speech. The present data reveal that four of the six speakers had a smaller vowel space area when the stimulation was on. This suggests that articulator mobility was reduced by stimulation. Some authors have suggested that current spread from the STN to the nearby fiber tracts may account for negative side effects in DBS, such as mild spasticity which is uncharacteristic of hypokinetic dysarthria [36]. Without further testing and stimulator adjustment, it cannot be known whether this occurred in the present study.

### 4.7. Perceptual Ratings

While previous studies have reported both improvements and decrements in speech with DBS, the goal with the present group of speakers was to learn more about the specific aspects of speech that were affected by stimulation, since the neurology staff had reported that these individuals' speech was worse with DBS than without. The judges' perceptual ratings revealed generally poorer speech in the on condition and thus were consistent with the participants' original referral to the study. 

It is notable that a number of the acoustic variables for some speakers showed improvement even though the perceptual ratings reflected the opposite. The acoustic measures used in the present study were selected because previous work has suggested that they might reveal which speech subsystems contribute to the perception of speech deterioration with DBS. For example, the perturbation and LTAS variables are associated with vocal fold activity and thus would be expected to show whether DBS affects the function of the larynx. Although perceived speech severity was the focus of the present listening task, the contribution of phonation to intelligibility in PD has been discussed previously by Ramig [37]. Consonant imprecision is a feature of most dysarthria subtypes [38], and it was reasoned that the index of spirantization would be sensitive to changes in this feature of dysarthria that is often reported in PD [19]. Likewise, the formant slope and vowel space area metrics were used because they are associated with lingual activity for the vocalic aspects of speech. However, because of the global nature of the perceptual rating made by our listeners, it is not possible to determine exactly which acoustic parameter may have been most responsible for the perceived deficits accompanying DBS.

The challenge of establishing a clear linkage between objective measures of speech and perceptual ratings is not new [26, 27]. Thus, it ought not to be surprising that the acoustic and human perceptual data in the present study included discrepancies. Speakers M5 and M10 tended to have a greater number of positive changes in the objective measures during stimulation. M5 was rated as having the most severe dysarthria and yet still showed several acoustic improvements with stimulation, although many of these were modest in scale. Because M10 was rated by the judges as having normal speech in both stimulation conditions, it would not be anticipated that his acoustic measures would change in a particular direction with DBS. Given this speaker's near-normal perceptual ratings, it is surprising that his acoustic measures were often worse than for the other speakers. This dissociation between acoustic and perceptual measures adds to the challenge in interpreting the overall effect of DBS on speech. 

While the measurement of percent intelligibility was beyond the scope of the present study, recent work from Tripoliti et al. [39] has shown that STN-DBS can lead to significant declines in direct measures of speech intelligibility, even when speech intensity increases. Future studies that examine such changes in relation to specific acoustic or physiologic speech measures would further our understanding of the mechanisms responsible for poorer speech performance. 

### 4.8. General Discussion

The results of the present study showed variability in the effect of DBS on participants' speech; some showed slight improvements with stimulation while others, particularly participant M9, performed markedly worse. The findings may have been influenced by the fact that the dysarthria of some participants was very mild without stimulation, and thus there may not have been much latitude for change with stimulation. 

Another finding that has been reported previously is that improvements or deterioration in the performance of one speech subsystem do not necessarily accompany similar change in another component of the speech mechanism [13]. In other words, it is possible for phonation to become worse while articulation improves in the same individual when the stimulation is on. Another observation from the same authors could be equally applied to the present study, namely, that it is not possible to make global statements about the effects of DBS on phonation or articulation because of the degree of interspeaker variability in their response. Similar findings of variability, as well as response differences linked to task effects, have been reported by others [40]. Hammer and colleagues [33] reported considerable heterogeneity in the response of a group of 18 individuals with PD to STN stimulation. Tripoliti and colleagues noted that patients who had electrodes placed more medially within the STN were more prone to speech deficits at higher stimulation voltages [41]. Since precise anatomic data were not available for the present study, it could be speculated that some of the inter-speaker differences may be attributable to slight differences in the location of the electrodes, in addition to individual stimulation settings.

In the present study the patients were all evaluated in a medicated state in order to simplify the interpretation of the effects of turning the DBS on or off. However, numerous studies have investigated the impact of levodopa on speech and the findings have been mixed. Skodda and colleagues [42] found no significant changes in several acoustic measures of speech in response to short- or long-term levodopa administration. Likewise, Plowman-Prine et al. [43] conducted a detailed perceptual evaluation of 35 speech dimensions and found no significant differences between the on and off medicated states. Ho et al. [44] reported that speech rate and intensity increased with levodopa use and suggested that these changes may parallel the typical limb motor improvements in speed and extent. However, the failure to maintain loudness across an utterance resulted in diminished overall speech benefit. On the other hand, De Letter and colleagues have reported significant improvements in word intelligibility [45] as well as positive changes in prosody and comprehensibility [46]. They recently evaluated the course of speech changes at multiple time points across a medication cycle and cautioned that it may be unwise to draw conclusions about the impact of medication based on a single assessment after patients take the drug [47]. This latter study in particular suggests that future investigations of the impact of DBS on speech should not ignore the potential time-varying medication effects that may complicate the interpretation of on/off DBS changes. Experimental protocols like that used in the present study may also be subject to the influence of fatigue when patients are tested under stimulation conditions that are separated by relatively long periods to “wash out” any residual stimulation effect.

### 4.9. Limitations of the Present Study

One limitation in the present study was the small number of participants, thus making it impossible to undertake inferential statistical analysis to determine the significance of the findings and to allow for generalization to a larger group of patients. Therefore, it would be beneficial for future research in this population to be conducted with a larger sample to allow an objective evaluation of the significance of the results. 

The lack of limb and axial motor data, both prior to surgery and in response to DBS, must be considered a significant limitation of the present investigation. Since patients are usually referred for surgery on the basis of their motor impairment and since these symptoms are generally the most responsive to DBS, it would be informative to consider changes in UPDRS scores before and after surgery. These changes, as well as differences between the on- and off-stimulation conditions, would provide a valuable context within which to evaluate the detailed speech acoustic measures. Following DBS patients are often able to significantly reduce their levodopa dosage, and since this may also influence speech performance, future work should consider this potential influence. Furthermore, detailed knowledge of the anatomic location of the electrodes and the specific stimulation parameters may increase our understanding of individual speaker differences in response to DBS.

Another limitation of the present work was that neither perceptual nor acoustic presurgical speech severity measures were available. Since the patients were only referred by the neurology staff on the basis of poorer speech on than off-stimulation, the investigators did not have access to the patients prior to the implantation of the electrodes. Thus, changes in speech related to electrode implantation microlesion effects as well as stimulation could not be tested within the context of the current study. It would have been valuable to learn whether any preexisting dysarthria was worsened by DBS or whether patients with normal speech before DBS became dysarthric following the surgery. Future work which records the speech of all DBS candidates at a medical facility prior to surgery would allow a clearer evaluation of the wider pre/postsurgical effects on speech. Such information would be clinically relevant as it could provide Parkinson's patients who are considering DBS as a treatment option with a better understanding of the possible speech-related consequences of surgery.

The perceptual rating task in the current study was limited to a global judgment of speech quality by relatively inexperienced listeners. Future work would benefit from a finer-grained perceptual assessment of speech characteristics by clinicians experienced with neuromotor speech disorders. This would allow the evaluation of multiple indices of speech quality (dysphonia type and severity, specific articulatory features, resonance changes, etc.) and a comparison of these with the acoustic measures. 

### 4.10. Conclusions

As it is possible that some individuals who opt for implantation will exhibit worsened speech with stimulation, it is important for neurology staff responsible for programming the stimulators after surgery to find the best possible balance between motor benefits and speech impairment to allow for the greatest quality of life. Recent work has shown that subtle differences in the exact anatomic placement of the electrodes and also the stimulation parameters (voltage, frequency, pulse width, etc.) can differentially influence speech and limb outcomes [48]. Other recent work with model-based rather than trial and error clinical programming of the stimulation parameters [49] may pave the way for improved programming that yields the maximal motor benefits while limiting the speech-related side effects.

## Figures and Tables

**Table 1:** Demographic data from the study participants.

Participant	Gender	Age	Years after Diagnosis	Medications
F1	F	79	24	Carbidopa/levodopa Mirapex
M4	M	56	4	Carbidopa/levodopa
M5	M	50	18	Carbidopa/levodopa
M8	M	54	15	Carbidopa/levodopa
M9	M	72	12	Carbidopa/levodopa
M10	M	48	10	Carbidopa/levodopa Mirapex

**Table 2:** Lenient (proper names included) and strict (proper names excluded) counts of verbal fluency with and without stimulation.

Participant	Count	Stim on	Letter	Stim off	Letter
F1	Strict	5	r	9	p
	Lenient	5	r	9	p
M4	Strict	9	p	10	r
	Lenient	9	p	12	r
M5	Strict	1	w	4	p
	Lenient	4	w	5	p
M8	Strict	9	r	16	p
	Lenient	11	r	22	p
M9	Strict	8	r	6	p
	Lenient	8	r	7	p
M10	Strict	8	w	4	r
	Lenient	9	w	6	r

**Table 3:** Vocal perturbation for vowel phonation with and without stimulation.

Participant	Variable	Stimulation on	Stimulation off
F1	Jitter (%)	7.69	2.35
	Shimmer (%)	5.69	5.79
	HNR (dB)	10.12	19.46
M4	Jitter (%)	2.22	1.17
	Shimmer (%)	16.57	8.52
	HNR (dB)	14.01	18.06
M5	Jitter (%)	0.43	0.44
	Shimmer (%)	1.24	5.15
	HNR (dB)	24.44	21.12
M8	Jitter (%)	0.22	0.27
	Shimmer (%)	0.80	0.77
	HNR (dB)	27.33	31.49
M9	Jitter (%)	0.58	0.62
	Shimmer (%)	5.60	6.66
	HNR (dB)	20.33	19.23
M10	Jitter (%)	1.53	2.59
	Shimmer (%)	8.36	12.78
	HNR (dB)	16.57	13.37

HNR: harmonics-to-noise ratio; higher values reflect better performance. Higher jitter and shimmer percentages are reflective of greater perturbation or vocal unsteadiness.

**Table 4:** Spectral moments (*M* and SD) of the long-term average spectrum for reading with and without stimulation.

Participant				
	Stimulation on		Stimulation off	
	*M* (kHz)	SD	*M *(kHz)	SD
F1	6.55	4.80	7.01	4.56
M4	5.07	5.20	4.23	4.61
M5	7.72	5.29	7.34	5.30
M8	4.19	4.77	4.71	4.60
M9	4.03	5.56	6.46	5.93
M10	7.49	3.29	8.18	2.78

**Table 5:** Ratios of mean (and standard deviation) vowel RMS to mean stop closure RMS as an index of spirantization with and without stimulation.

Participant	Syllable	Ratio stim on	Ratio stim off
F1	/p*ə*/	3.43 : 1	1.86 : 1
	/t*ə*/	9.60 : 1	5.50 : 1
	/k*ə*/	4.30 : 1	3.00 : 1
M4	/p*ə*/	13.67 : 1	12.50 : 1
	/t*ə*/	16.67 : 1	11.50 : 1
	/k*ə*/	10.50 : 1	6.25 : 1
M5	/p*ə*/	2.93 : 1	3.69 : 1
	/t*ə*/	9.25 : 1	9.15 : 1
	/k*ə*/	5.00 : 1	3.91 : 1
M8	/p*ə*/	31.00 : 1	18.00 : 1
	/t*ə*/	16.00 : 1	19.50 : 1
	/k*ə*/	12.50 : 1	13.50 : 1
M9	/p*ə*/	2.56 : 1	22.75 : 1
	/t*ə*/	3.59 : 1	12.20 : 1
	/k*ə*/	1.77 : 1	11.50 : 1
M10	/p*ə*/	7.89 : 1	7.60 : 1
	/t*ə*/	7.43 : 1	6.00 : 1
	/k*ə*/	6.86 : 1	5.20 : 1

A higher value for the ratio reflects less severe spirantization.

**Table 6:** Average (and standard deviation) F1 and F2 slope (Hz/ms) for the diphthongs with and without stimulation.

Participant	Diphthong	Stimulation on		Stimulation off	
		F1	F2	F1	F2
F1	/*ɔɪ*/	−0.74 (0.72)	11.07 (2.14)	−0.60 (0.81)	9.26 (2.46)
	/*ɑʊ*/	−0.94 (0.30)	−0.85 (0.35)	−0.55 (0.64)	−0.34 (1.02)
	/*ɑɪ*/	−0.15 (0.44)	2.98 (1.15)	−0.86 (0.61)	2.61 (0.77)
	/e*ɪ*/	−2.31 (1.71)	4.81 (0.94)	−2.64 (1.24)	2.69 (0.53)
M4	/*ɔɪ*/	−0.24 (0.37)	6.45 (0.65)	−0.24 (0.62)	7.82 (1.40)
	/*ɑʊ*/	0.32 (0.73)	−3.87 (0.74)	0.02 (0.26)	−2.79 (0.38)
	/*ɑɪ*/	−0.78 (0.35)	3.35 (1.43)	−0.97 (0.28)	2.59 (0.85)
	/e*ɪ*/	−0.87 (0.52)	0.95 (0.72)	−0.68 (0.65)	1.54 (0.58)
M5	/*ɔɪ*/	−0.68 (0.73)	9.79 (2.07)	−0.08 (0.75)	8.62 (2.16)
	/*ɑʊ*/	0.37 (0.46)	−3.19 (0.31)	0.21 (0.24)	−2.15 (0.66)
	/*ɑɪ*/	−1.53 (0.64)	3.22 (0.81)	−1.57 (0.65)	3.61 (0.43)
	/e*ɪ*/	−0.45 (0.53)	2.02 (0.95)	−0.10 (0.30)	2.63 (1.48)
M8	/*ɔɪ*/	−0.88 (0.42)	8.46 (0.54)	−0.10 (0.29)	8.58 (0.81)
	/*ɑʊ*/	0.57 (0.63)	−1.86 (0.62)	0.69 (0.69)	−2.43 (0.66)
	/*ɑɪ*/	−0.60 (0.88)	2.46 (0.42)	−1.23 (0.33)	2.54 (0.68)
	/e*ɪ*/	−0.84 (0.27)	1.44 (0.28)	−0.49 (0.22)	0.94 (0.28)
M9	/*ɔɪ*/	−0.80 (0.53)	9.47 (1.92)	−1.21 (0.83)	9.64 (2.10)
	/*ɑʊ*/	−0.32 (0.52)	−2.16 (0.77)	0.29 (0.60)	−1.92 (1.21)
	/*ɑɪ*/	−0.62 (1.30)	2.62 (1.94)	−0.86 (0.23)	1.50 (2.64)
	/e*ɪ*/	−0.69 (0.73)	1.72 (0.66)	−1.18 (0.84)	2.01 (0.14)
M10	/*ɔɪ*/	−1.00 (0.92)	11.50 (2.10)	−0.79 (0.74)	10.86 (1.87)
	/*ɑʊ*/	0.23 (0.34)	−2.65 (0.89)	0.49 (0.52)	−3.12 (0.57)
	/*ɑɪ*/	−0.49 (0.51)	4.44 (1.17)	−0.49 (1.26)	2.85 (0.94)
	/e*ɪ*/	−0.83 (0.60)	1.17 (0.55)	−0.83 (0.31)	−0.06 (0.83)

**Table 7:** Vowel space area (Hz^2^) computed from the four corner vowels with and without stimulation.

Participant	Stimulation on	Stimulation off
F1	102240	105160
M4	237710	290990
M5	152260	118610
M8	176020	204370
M9	116240	239960
M10	168950	139880

**Table 8:** Perceptual ratings of the reading passage and acoustic analysis sentences with and without stimulation.

	Reading		Sentences	
	Stimulation on	Stimulation off	Stimulation on	Stimulation off
F1	62.3	52.1	49.1	46.1
M4	20.8	10.9	36.7	31.1
M5	80.4	68.9	68.8	65.8
M8	30.8	15.4	10.1	37.0
M9	53.6	34.7	60.3	18.9
M10	0.0	0.9	1.8	1.7

Note: High scores reflect greater dysarthria severity (0 = normal, 100 = severely dysarthric).
